# Life-span extension by pigmented rice bran in the model yeast *Saccharomyces cerevisiae*

**DOI:** 10.1038/s41598-019-54448-9

**Published:** 2019-12-02

**Authors:** Pitchapat Sunthonkun, Rinsai Palajai, Pichayada Somboon, Chua Lee Suan, Malyn Ungsurangsri, Nitnipa Soontorngun

**Affiliations:** 1 Division of Biochemical Technology, School of Bioresources and Technology, King Mongkut’s University of Technology Thonburi, Bangkok, 10150 Thailand; 2 Metabolites Profiling Laboratory, Institute of Bioproduct Development, Universiti Teknologi Malaysia, 81310 UTM Skudai, Johor Bahru, Johor Malaysia; 3 Research and Development division, S&J International Enterprises Public Company Limited, Bangkok, Thailand

**Keywords:** Senescence, Natural products

## Abstract

Benefits of whole grains as dietary supplements and active ingredients in health products have been promoted. Despite being neglected as an agricultural byproduct of polished rice, pigmented rice bran has emerged as a promising source of natural anti-aging compounds. Indeed, the extract of red rice bran Hom Dang cultivar contained rich phenolic acids and flavonoids. It displayed high antioxidant activities *in vitro* and *in vivo* assays. Using yeast model, extract and bioactive compounds, quercetin and protocatechuic acid found in the rice bran pericarp, effectively reduced levels of intracellular reactive oxygen species (ROS), restored plasma membrane damages and prolonged life-span of pre-treated wild-yeast cells. Importantly, these molecules modulated life span-extension through a mechanism of ROS reduction that resembles to that operated under the highly conserved Tor1- and Sir2-dependent signaling pathways, with the human homologs TORC1 and SIRT1, respectively. The key longevity factors Sch9 and Rim15 kinases, Msn2/4 regulators and a novel transcription factor Asg1, the antioxidant enzymes superoxide dismutases and glutathione peroxidases played important role in mediating longevity. Yeast clearly provides an instrumental platform for rapid screening of compounds with anti-aging efficacies and advances knowledge in the molecular study of ageing.

## Introduction

Due to its genetic simplicity, rapid growth, ease of cultivation, the yeast *Saccharomyces cerevisiae* has many useful applications including in winemaking, baking, and brewing^1,2^. *S*. *cerevisiae* has a well-characterized genome with conserved genetic pathways that resemble those of mammalian eukaryotic cells, which makes it a eukaryotic model of choice for gaining insights into the molecular and cellular biology of higher organisms, including humans. Particularly, it is a useful model to study cellular responses to oxidants owing to several conserved antioxidant enzymes. *S*. *cerevisiae* produces endogenous reactive oxygen species (ROS) via physiological respiration of electron transport chain activity in mitochondria^3^. Exogenous sources of ROS include UV light, air pollution, inflammation and ionizing radiation^4,5^. ROS/RNS consist of free radicals such as superoxide (O_2_^•−^), peroxynitrite (ONOO^•−^), hydroxyl (OH^•^), and non-radicals such as hydrogen peroxide (H_2_O_2_)^6^. Oxidative stress results from an imbalance between oxidants, such as ROS, and antioxidants in favor of the oxidants, leading to macromolecular damages of proteins, lipids, and/or DNA^7^, which could trigger apoptotic cell death^8^. Several reports have revealed that accumulation of ROS leads to the development of human diseases such as cancer, brain (Parkinson, Alzheimer’s and migraine), cardiovascular, and inflammatory diseases^4^.

Antioxidant molecules are commonly present in the form of natural plant extracts^9^. Rice (*Oryza sativa* L.) is one of the most important grain foods consumed worldwide and contains a range of bioactive compounds with antioxidant potential^10,11^.The amount of antioxidants found in rice, however, differs depending on grain fractions, e.g. whole grain, bran, husk, germ and endosperm, grain colors, grain types, e.g. Catahoula, Cheniere, Caffey, and the extraction processes^12^. Most phytochemicals, such as tocopherols, tocotrienols (vitamin E) and γ-oryzanol, in cereals are present in the bran (containing the pericarp tissue and aleurone layer) and germ fractions, and are available in lipophilic, hydrophobic and insoluble forms with potential health benefits^13,14^. The USDA National Small Grains Collection (NSGC) has classified rice bran by color into seven classes: white, light-brown, speckled brown, brown, red, variable purple, and purple. Anthocyanins, proanthocyanins and many other phenolic compounds have been reported in red and black rice varieties^15^, in addition to flavonoids including flavonols, flavan-3-ols, flavones, and flavanones^16^. Phenolic compounds such as ferulic acid, *p*-coumaric acid, vanillic acid, caffeic acid, syringic acid, and sinapinic acid have also been found in some varieties of black rice^17^. The most abundant phenolic acids in rice bran of black and red rice are ferulic acid and *p*-coumaric acid, which accounts for almost 50–65% of total phenolic acids^18,19^. In addition, the flavonoids include flavonols, flavan-3-ols, flavones and flavanones are reported^16^. High amount of phytochemicals with antioxidant capacity have a wide range of therapeutic effects against chronic diseases^20,21^.

In *S*. *cerevisiae*, biological aging is evolutionarily conserved and controlled by a network of proteins kinases in many signaling pathways. The network of chronological aging integrates the pro-aging TORC1 (target of rapamycin complex pathway), which stimulates the pro-aging protein kinase Sch9, and pro-aging SIR. In contrast, it includes antagonist mechanisms including the anti-aging Snf1 (sucrose non-fermenting) pathway, the anti-aging ATG (autophagy) pathway, and the anti-aging protein kinase Rim15, inhibited by the TORC1, PKA, and PKH1/2 pathways. The protein kinase Rim15 phosphorylates Msn2/4 transcription factors activate gene expression by binding to the stress response element (STRE) to control environmental stresses, including heat shock, oxidative stress, and nutrient starvation. Msn2/4 transcription factors modulate manganese superoxide dismutase encoding-*SOD2* genes in defense against toxic ROSs^22^. Two SODs, namely Cu-Zn dependent cytosolic and intermembrane space Sod1 and Mn-dependent Sod2, which are localized it the mitochondrial matrix guard against mitochondrial superoxide production^20–23^. In addition, *S*. *cerevisiae* has glutathione peroxidases to protect cells from oxidative damage^23^. *GPX1* gene encodes an enzyme, Gpx1, that functions in the detoxification of hydrogen peroxide particularly by catalyzing the reduction of hydrogen peroxide to water^23^. *GPX2* gene encodes an atypical 2-Cys peroxiredoxin or phospholipid hydroperoxide glutathione peroxidase that applies thioredoxin as an electron donor^24^. In aerobic metabolism, antioxidant functions of the reduced glutathione GSH can be metabolized by glutathione peroxidases (Gpx) in the cytosol and mitochondria^23,25^.

Despite extensive studies on the existence of antioxidants in pigmented rice brans, little is known regarding the role they play in longevity. The aims of this study were to apply the yeast *S*. *cerevisiae* gene knockout system to identify new longevity factors and to determine the antioxidant and anti-aging potential of pigmented rice bran extract and compounds with the focus on their beneficial effect on chronological lifespan. Some candidates for anti-aging drugs, such as spermidine and resveratrol, and anti-aging interventions, such as caloric restriction, have been identified and explored in yeast^26^. Insights into the involved mechanism will help us to further explore other potential anti-aging agents.

## Results

### Total phenolic content (TPC) and antioxidant activities of pigmented rice bran extract

The antioxidant activities of bran extracts from two pigmented rice varieties (red and black) were determined by *in vitro* assays. These included the inhibition of 2, 2-diphenyl-1-picrylhydrazyl (DPPH) using 2,2′-azino-bis-3 ethylbenzthiazoline-6-sulphonic acids (ABTS) as an oxidant and FRAP (Ferric reducing antioxidant power). Total phenolic contents were also determined. The extraction was performed using two polar solvents, ethanol and propylene glycol, separately. The TPC of ethanolic rice bran extracts of Hom Dang (red rice) and Kum Doi Saket (black rice) was in the range of 1.20 ± 0.16 to 1.69 ± 0.12 mg GAE/g extract, while propylene glycolic extracts ranged from 1.09 ± 0.06 to 1.44 ± 0.08 mg GAE/g extract (Table 1). Of both rice varieties, the highest TPC was obtained from Hom Dang rice bran ethanolic extract (Table 1). Its DPPH free radical scavenging activity also showed the lowest EC_50_ (0.35 ± 0.20 mg/mL). The ABTS scavenging assays also showed similar antioxidant capacity for all rice bran extracts tested (Table 1). Also, the propylene glycolic extract of Kum Doi Saket had the highest ferrous-chelating capacity (FRAP) of 7.29 ± 0.50 × 10^6^ µmole Fe(II)/g extract followed by the ethanolic Hom Dang rice bran and Kum Doi Saket rice bran extracts (Table 1). High total anthocyanin content involved in high ferrous-chelating capacity (FRAP), in the black rice bran extract is proposed to be responsible for the antioxidant activity among other pigmented rice bran extract. Overall, Hom Dang rice bran extract showed the best TPC and scavenging activity (Table 1).

**Table 1:** *In vitro* antioxidant properties of Thai pigmented rice bran extracts.

Rice bran	^a^TPC	^b^EC_50_ (mg/mL)		^e^FRAP
Extraction	(mg GAE/g DW)	^c^DPPH	^d^ABTS	(µmoleFe(II)/g extract)
**Propylene Glycol**				
Hom Dang	1.44 ± 0.08	0.47 ± 0.25	0.006 ± 0.001	5.85 ± 0.20 × 10^6^
Kum Doi Saket	1.09 ± 0.06	0.59 ± 0.09	0.008 ± 0.002	7.29 ± 0.50 × 10^6^*
**Ethanolic**				
Hom Dang	1.69 ± 0.12	0.35 ± 0.20	0.007 ± 0.002*	6.85 ± 1.30 × 10^6^
Kum Doi Saket	1.20 ± 0.16	0.45 ± 0.50	0.013 ± 0.012	6.39 ± 0.60 × 10^6^
Ascorbic acid		0.02 ± 0.13*	N.I	N.I

^a^Total phenolic content in milligram of gallic acid in 1 g of pigmented rice bran extract.

^b^EC_50_= the concentration of a compound that gives half-maximal response.

^c^% Inhibition of 2,2-Diphenyl-1-picrylhydrazyl radical scavenging activity.

^d^50% 2,2′-azino-bis-3-ethylbenzthiazoline-6-sulphonic acid scavenging activity.

^e^Ferric reducing/antioxidant power in milligram of Trolox equivalent in 1 g of pigmented rice bran extract.

^f^N.I = Not identified.

Values are presented as means ± S.D. Statistical significance was considered at: *p < 0.01.

### Determination of antioxidant compounds of pigmented rice bran extract

The UV spectra of the hydroxybenzoic acids were relevant and used to elucidate their chemical structures (Table 2). Single absorption peaks appeared in the UV spectra of compounds such as *p*-coumaric acid providing a [M-H]^−^ anion at m/z 119 and protocatchuic acid providing a [M-H]^−^ anion at m/z 109, which have symmetrical chemical structures. However, the position and number of hydroxyl groups on the aromatic rings also had a significant effect on wavelength shift. The ferulic acid provided [M-H]^−^ anions at m/z 134, m/z 149, m/z 178, all of which had non-symmetrical chemical structures, a major absorption peak and a shoulder absorption under our conditions, which was inconsistent with the previous report^16,24^. (+)-Catechin ([M-H]^−^) yielded fragment ions at m/z 205 and m/z 245, which were produced by the loss of a (CH)_2_OH group. Also, (−)ESI-MS/MS spectra of the procyanidin dimer and procyanidintrimer ([M-H]^−^ gave fragment ions at m/z 695 from rearrangement of the heterocyclic ring^27^, at m/z 425 [M-H]^−^ from RDA-F of the heterocyclic ring and loss of H_2_O, at m/z 289 [M-H]^−^ from cleavages between C_4_-C_5_ and O-C_2_ of one pyran ring, and at m/z 289 ([M-H-289]^−^) from cleavage of the interflavanic bond. Kaempferol had a fragment ion at m/z 257, m/z 151, m/z 169, also from the cleavage of the heterocyclic C-ring by RDA, but the fragmentation mechanism remains unclear at present. For flavonol-O-glycosides such as rutin fragment ions at m/z 301, m/z 179, m/z 151, and quercetin at m/z 151, m/z 179, their spectra showed a deprotonated [M-H]^−^ molecule of the glycoside and an [A-H]^−^ ion corresponding to the deprotonated aglycone. The latter ion is formed by losing the rutinose moiety from the corresponding glycosides.

**Table 2:** Analysis of bioactive compounds in pigmented rice bran extracts of Hom Dang rice bran (HD) and Kum Doi Saket rice bran (KD).

Identified Compounds	Structures	Rt (min)	[M-H]^-^	Rice bran cultivars
**Phenolic acids**				
*p*-Courmaric acid		7.59	119	HD,KD
Protocatchuic acid		5.66	109	HD,KD
Ferulic acid		7.91	134,149,178	HD, KD
**Flavonoid**				
Proanthocyanidin dimer		6.30	425,289	HD
Proanthocyanidin trimer		6.41	695	HD
Catechin		6.41	245,205	HD
Rutin		7.93	301,179,151	HD,KD
Kaemferol		8.99	257,151,169	HD,KD
Isorhamnetin		10.26	300	HD,KD
Quercetin		9.51	151,179	HD
α-tocopherol		5.43	431,343,205	HD,KD

In summary, the ESI-MS/MS spectra analyzed showed that propylene glycolic Hom Dang rice bran extract contained many free phenolic acids including *p*-courmaric acid, protocatchuic acid, and ferulic acid (Table 2). It also contained tannin, including proanthocyanidin dimer and proanthocyanidin trimer, as well as α-tocopherol and flavonoids, including catechin, rutin, kaempherol, isorhamnetin, and quercetin (Table 2). However, the propylene glycolic extract of Kum Doi Saket contained less bioactive compounds, only ferulic, isorhamnetin, protocatechuic acid, rutin, kaempherol, and p-courmaric acid (Table 2). Based on the LC/MS-MS analysis, Hom Dang rice bran extract contained, in comparison, a higher amount of bioactive compounds, some of which included phenolic acids and flavonoids.

### Effects of pigmented rice bran extracts on H_2_O_2_ scavenging

*S*. *cerevisiae* is a good and easy organism for use as an *in vivo* model for testing the ability of antioxidants to scavenge intracellular ROS^28^. It was used to investigate the ability of Hom Dang rice bran extract to scavenge intracellular ROS generated by the potent oxidant H_2_O_2_, based on the DCF method^29^. The conversion of the non-fluorescent DCFH_2_-DA to highly fluorescent DCF is well documented and occurs in several steps. DCFH_2_-DA is transported across the cell membrane and deacetylated by esterases to form the non-fluorescent DCFH_2_. This compound is trapped inside the cells. Next, DCFH_2_ is converted to DCF through the action of intracellular oxidants; H_2_O_2_, HO^•^, ROO^•^ ^30^. Different yeast deletion strains lacking key genes in aging and antioxidant systems, namely *SOD1*, *SOD2*, *GPX1*,*GPX2*, *TOR1*, *SIR2*, *RIM15*, *MSN2/4*, *ASG1*, and *SCH9*, were pre-treated with Hom Dang rice bran extracts prior to the exposure to 5 mM H_2_O_2_.

**Figure 1:**
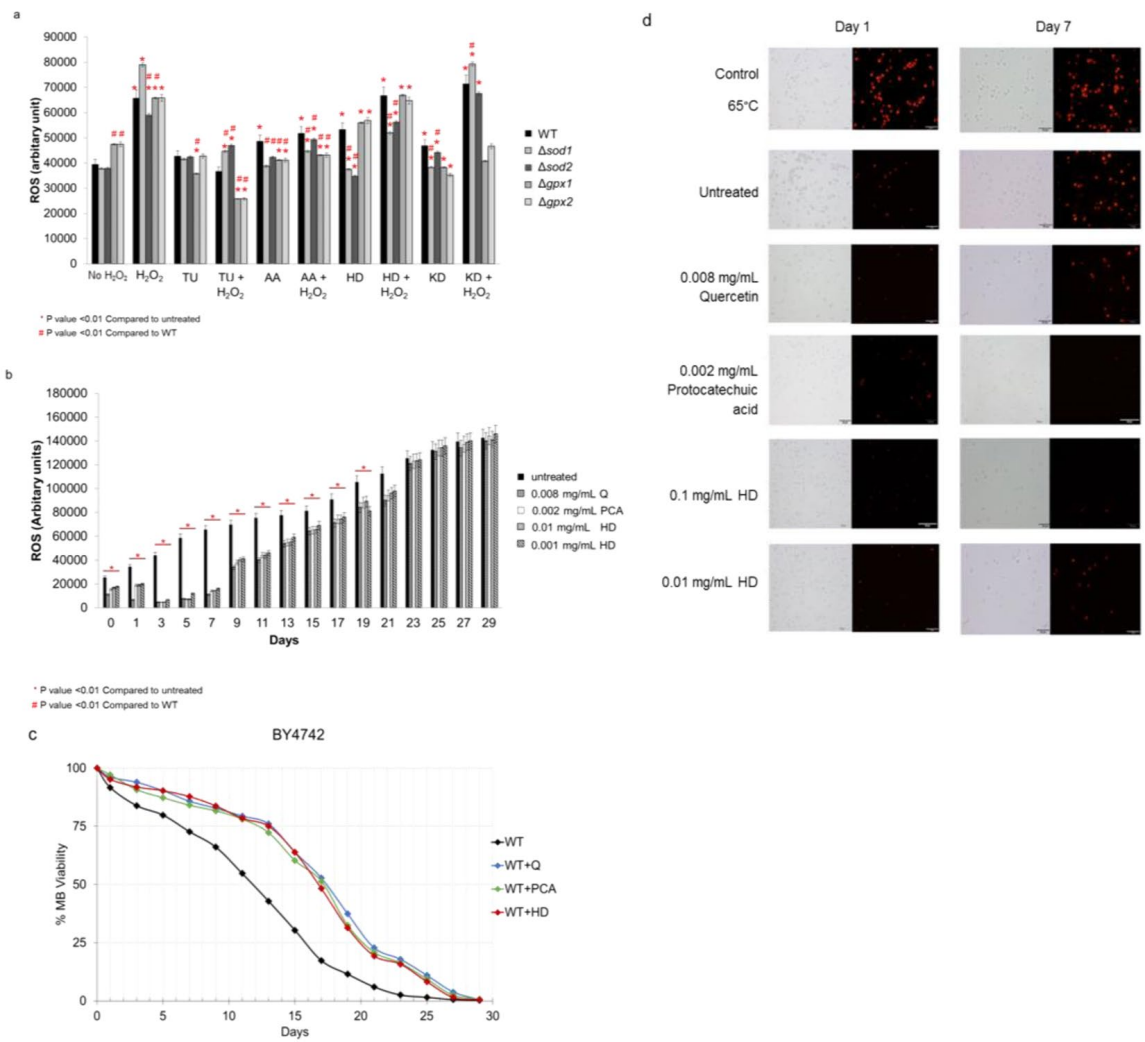
(**a**) Quantification of cellular ROS levels via the fluorescent DCFH-DA assay in the *S*. *cerevisiae* wild-type (WT) and mutant yeast cells. The WT, the ∆*sod1* and the ∆*sod2* strains were treated with 0.01 mg/mL Hom Dang rice bran extract (HD). The WT the ∆*gpx1* and the ∆*gpx2* strains were treated with 0.01 mg/mL Kum Doi Saket rice bran extract (KD). After, cells were exposed to hydrogen peroxide (H_2_O_2_) to induce generation of ROS. Endogenous ROS levels of cells were measured by a fluorometric assay using 2′,7′-dichlorofluorescein diacetate (DCFH-DA) as a ROS indicator. Error bars represented standard error of the mean (SEM) (*p, ^#^*p* < 0.01, two-tailed Student’s t test compared to untreated condition and wild-type, respectively). TU referred to thiourea and AA referred to ascorbic acid. (**b**) Quantification of intracellular ROS levels in the chronological ageing *S*. *cerevisiae* WT strain treated with antioxidants 0.008 mg/mL of quercetin (Q), 0.002 mg/mL of protocatechuic acid (PCA), 0.1 or 0.01 mg/mL Hom Dang rice bran extract (HD) for 29 days. The ROS levels were analyzed using the fluorescent probe DCFH-DA. Error bars represented standard error of the mean (SEM) (*p < 0.01, two-tailed Student’s t test compared to untreated condition). (**c**) Survival curves of chronologically aging yeast cells from day 0 to day 30 were shown for the wild-type *S*. *cerevisiae* BY4742. Pre-treatment with antioxidants quercetin (Q) or protocatechuic acid (PCA) or Hom Dang rice bran extract (HD) showed increasing life-span extension only in the WT strain. (*p < 0.01, two-tailed Student’s t test compared to untreated condition). (**d**) Plasma membrane permeability of the ageing *S*. *cerevisiae* WT strain at day 1 and day 7 was examined. The WT strain was either chronologically grown at 30 °C without antioxidants (untreated negative control), or treated with antioxidants 0.008 mg/mL of quercetin (Q), 0.002 mg/mL of protocatechuic acid (PCA), 0.1 or 0.01 mg/mL Hom Dang rice bran extract (HD) or heated to 65 °C for 10 min. (positive control). Cells were stained with propidium iodide and, the membrane permeability was analyzed by fluorescence microscopy.

The DCFH-DA assay showed that the ROS levels were increased after exposure to 5 mM H_2_O_2_ for all strains, especially the Δ*sod1* strain lacking the enzyme superoxide dismutase, which showed a 2-fold increase in the ROS level (Fig. 1a). Addition of the antioxidant agents, thiourea (TU) or ascorbic acid (AA or vitamin c), did not alter the levels of ROS but contributed to the lowering of the ROS level. As shown, the ROS levels dropped to the same or lower level than under untreated (H_2_O_2_) condition for all tested strains (Fig. 1a). The ROS levels in the WT, the Δ*sod1* and the ∆*sod2* strains were significantly decreased to the background ROS level after treatment with 0.01 mg/mL of the Hom Dang rice bran extract (Fig. 1a). Similarly, ROS levels in the WT, the ∆*gpx1*, and the ∆*gpx2* strains were significantly decreased after treatment with 0.01 mg/mL of the Kum Doi Saket rice bran extract (Fig. 1a). Thus, the results suggested the antioxidant activity of the pigmented rice bran extracts to reduce intracellular ROS levels in mutant yeast strains lacking antioxidant enzymes.

### Ability of Hom Dang rice bran extract to reduce intracellular ROS in aged yeast

Due to the high antioxidant activity of Hom Dang rice bran extract, its ability to reduce intracellular ROS was evaluated against two bioactive compounds, quercetin and protocatechuic acid, found in the rice bran (Table 2). The wild-type yeast *S*. *cerevisiae* strain was cultured for a total duration of 30 days. Addition of the Hom dang rice bran extract, quercetin or protocatechuic acid gradually decreased the intracellular fluorescence intensity from day 3 until day 21, when used at a concentration of 0.01–0.1, 0.008, and 0.002 mg/mL, respectively (Fig. 1b). The antioxidants could permeate through the yeast cell membrane and intercept ROS forming radicals, thereby preventing the production of different ROS compounds required to oxidize intracellular DCFH_2_ to the fluorescent DCF through post-diauxic and stationary phases (Fig. 1b). The efficient suppression of intracellular ROS production by the Hom dang rice bran extract, quercetin, and protocatechuic acid indicated that these agents act with strong radical scavenging potency in the intracellular environment.

### Hom Dang rice bran extract treatment prevented cell membrane damage

To provide additional evidence for the aforementioned findings, we examined the membrane permeability of the pigmented rice bran extract, quercetin or protocatechuic acid-pretreated *S*. *cerevisiae* cells using propidium iodide dye (Pi^+^)^31^. The exclusion of this hydrophilic red standard dye from cells with intact membranes is indicative if its integrity, whereas severely damaged cell membranes allow the dye in^32^. It is used to determine the membrane impermeability. Results showed that the reduction of Pi^+^ dye by the Hom Dang rice bran extract is time and concentration dependent. At day1, the untreated and pre-treated *S*. *cerevisiae* cells had low levels of fluorescence intensity, indicating minimal cell membrane damage (Fig. 1d). The fluorescence was also shown for the control, 65 °C-heated cells, at day 1 and day 7. By day 7 of culturing, Pi^+^ was strongly fluorescent in the untreated condition as cells aged (Fig. 1d). However, the fluorescence was dramatically reduced in cells treated with 0.1 mg/mL of the Hom Dang rice bran (HD) or 0.002 mg/mL of the protocatechuic acid (PCA) (Fig. 1d), which correlates well with low ROS levels of cells and is highly efficient against intracellular radicals (Fig. 1a–c). A lower concentration of (0.01 mg/mL) Hom Dang rice bran extract (HD) and 0.008 mg/mL of quercetin (Q) had less effect on membrane permeability and only protected yeast cells at day1 (Fig. 1d). Overall the results suggested that increased intracellular ROS accumulation in aged cells correlates with increased plasma membrane permeability (Fig. 1b,d). Similarly, decreased ROS accumulation in *S*. *cerevisiae* cells, when incubated with the Hom Dang rice bran extract (HD) and corresponding pure antioxidants, protocatechuic acid (PCA) and quercetin (Q), supported their mechanistic involvement in ROS scavenging and activation of stress response systems during the aging process.

### Antioxidants and Hom Dang rice bran extract extended lifespan of wild-type yeast cells

Aging has been associated with an increase in the accumulation of ROS and a decrease in antioxidant defenses. This oxidative damage theory of aging is supported by data showing that increased scavenging of ROS by over expression of antioxidant enzymes delays aging^31^. The protection against oxidative stress conferred by the antioxidants and the extract of Hom Dang rice bran extract led us to analyze their effect on yeast chronological life span. 0.1 mg/mL of Hom Dang rice bran extract increased the lifespan of *S*. *cerevisiae* wild-type BY4742 strain significantly compared to the untreated strain (Fig. 1c and Table 3). The life-span extension was increased by 5 days at 50% viability when compared to the untreated wild-type strain (Fig. 1c). Similarly, pre-treatment with 0.002 mg/mL of protocatechuic acid (PCA) or 0.008 mg/m of quercetin (Q) could extend the lifespan of the wild-type strain by 5 days at 50% viability compared to untreated strain (Fig. 1c). During chronological aging, the viability of cells pretreated with antioxidants Q or PCA or the extract showed positive effects, possibly due to the anti-oxidation potential of antioxidants. They may trigger the anti-aging system of yeast cells to gain better resistance to toxic or accumulated free radicals.

**Table 3:** % Normalized MB Viability and difference in MB Viability normalized to day 0, the untreated condition or WT of CLS assay at day 0, 7, 13, and 17 (*p < 0.0001). Q, PCA and HD referred to quercetin, protocathechuic acid and Hom Dang, respectively. Negative values indicated the decreased values.

Strain	Day	% Normalized MB Viability compared to day 0				% Difference in MB Viability normalized to day 0				% Difference in MB Viability normalized to untreated condition				% Difference in MB Viability normalized to WT			
		untreated	Quercetin (Q)	PCA	HD extract	untreated	Quercetin (Q)	PCA	HD extract	untreated	Quercetin (Q)	PCA	HD extract	untreated	Quercetin (Q)	PCA	HD extract
WT	0	100	100	100	100	0	0	0	0	0	0	0	0	0	0	0	0
	7	73	86	84	88	−27	−14	−16	−12	0	18*	16*	21*	0	0	0	0
	13	43	76	72	75	−57	−24	−28	−25	0	77*	69*	75*	0	0	0	0
	17	17	53	51	48	−83	−47	−49	−52	0	205*	197*	179*	0	0	0	0
∆*sir2*	0	100	100	100	100	0	0	0	0	0	0	0	0	0	0	0	0
	7	81	80	80	80	−19	−20	−20	−20	0	−1	−1	−2	12*	−7*	−5	−9
	13	63	63	65	63	−37	−37	−35	−37	0	1	4	1	46*	−17*	−10*	−16*
	17	29	28	26	28	−71	−72	−74	−72	0	−4	−7	−3	65*	−48*	−49*	−43*
∆*tor1*	0	100	100	100	100	0	0	0	0	0	0	0	0	0	0	0	0
	7	79	79	81	78	−21	−21	−19	−22	0	0	1	−2	9*	−8*	−4	−11
	13	60	57	62	61	−40	−43	−38	−40	0	−6	2	0	41*	−26*	−15*	−19*
	17	30	28	30	31	−70	−72	−70	−69	0	−7	−3	2	75*	−47*	−43*	−36*
∆*sch9*	0	100	100	100	100	0	0	0	0	0	0	0	0	0	0	0	0
	7	78	79	81	79	−22	−21	−19	−21	0	0	3	1	8*	−8*	−4	−10
	13	61	62	62	61	−39	−38	−38	−40	0	3	1	0	41*	−18*	−15*	−19*
	17	30	32	34	31	−70	−68	−66	−69	0	7	10	2	75*	−39*	−35*	−36*
∆*rim15*	0	100	100	100	100	0	0	0	0	0	0	0	0	0	0	0	0
	7	62	74	75	79	−38	−26	−25	−21	0	19*	20*	27*	−14*	−14*	−11*	−10*
	13	32	40	47	46	−68	−60	−53	−54	0	26*	46*	44*	−25*	−47*	−35*	−39*
	17	15	19	22	22	−85	−81	−78	−78	0	33*	53*	53*	−16*	−63*	−57*	−54*
∆*msn2*	0	100	100	100	100	0	0	0	0	0	0	0	0	0	0	0	0
	7	62	70	76	76	−38	−30	−24	−24	0	13*	22*	22*	−14*	−18*	−9*	−13*
	13	30	42	45	44	−70	−58	−55	−56	0	41*	52*	48*	−30*	−45*	−37*	−41*
	17	10	18	23	20	−90	−82	−77	−80	0	78*	127*	92*	−41*	−65*	−55*	−59*
∆*msn4*	0	100	100	100	100	0	0	0	0	0	0	0	0	0	0	0	0
	7	60	70	76	75	−40	−30	−24	−25	0	16*	26*	24*	−17*	−18*	−9*	−14*
	13	31	42	45	45	−69	−58	−55	−55	0	37*	47*	47*	−28*	−45*	−37*	−40*
	17	10	18	23	21	−90	−82	−77	−79	0	78*	127*	103*	−41*	−65*	−55*	−57*
∆*asg1*	0	100	100	100	100	0	0	0	0	0	0	0	0	0	0	0	0
	7	62	71	75	75	−38	−29	−25	−25	0	16*	22*	22*	−15*	−17*	−10*	−15*
	13	32	44	45	45	−68	−56	−55	−55	0	36*	41*	41*	−25*	−43*	−37*	−40*
	17	10	17	20	19	−90	−83	−80	−81	0	69*	98*	88*	−41*	−67*	−60*	−60*

### The Sir2 signaling pathway and the anti-aging effect of antioxidants and extract

**Figure 2:**
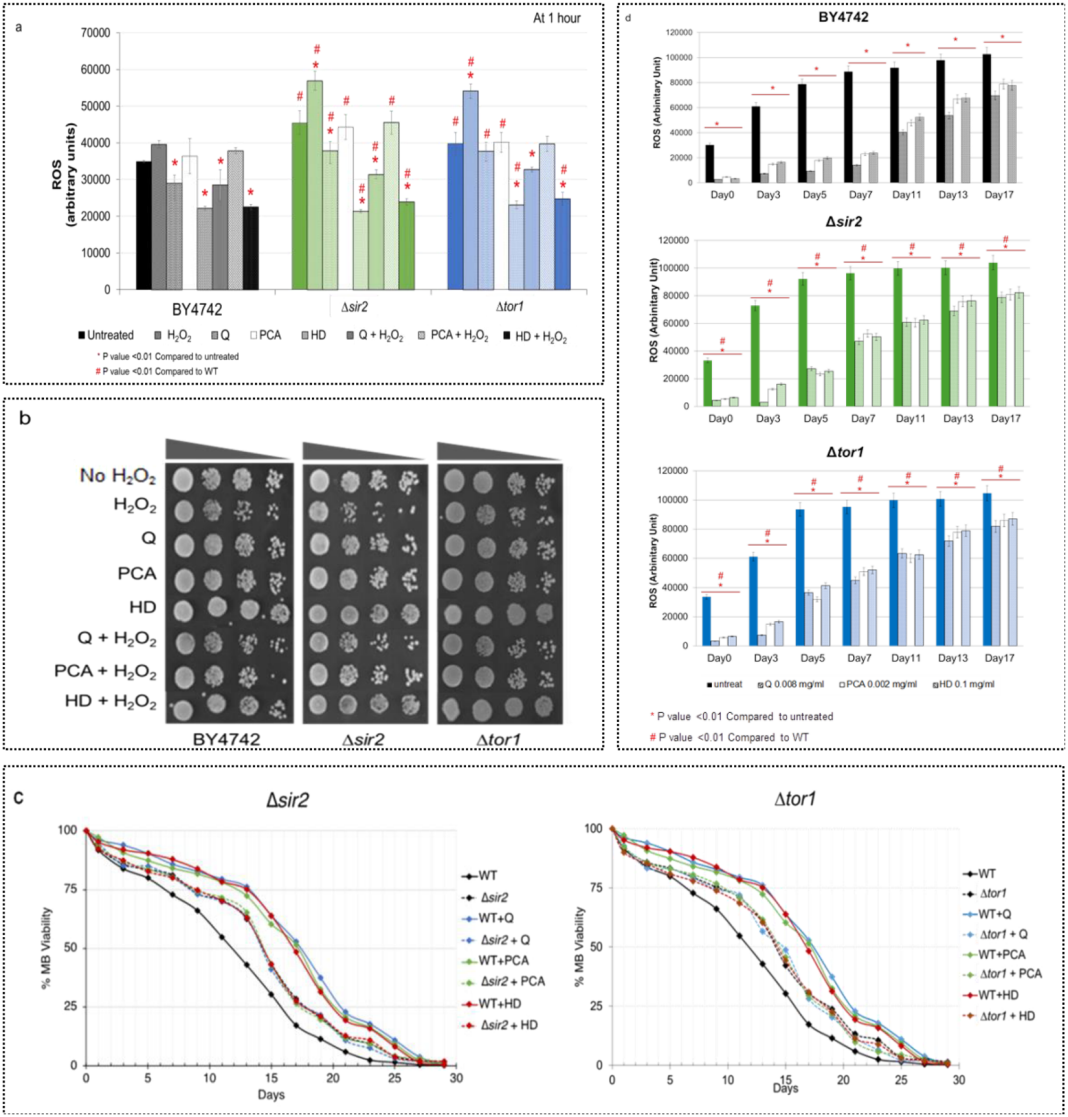
**(a**) Survival curves of chronologically aging yeast cells from day 0 to day 30 were shown. Lack of Tor1 or Sir2 significantly increased the longevity of *S*. *cerevisiae*. Pre-treatment with antioxidants quercetin (Q) or protocatechuic acid (PCA) or Hom Dang rice bran extract (HD) showed increasing life-span extension only in the WT strain but not in the deletion mutants of Tor1 or Sir2. (*p, ^#^*p* < 0.01, two-tailed Student’s t test compared to untreated condition and wild-type, respectively). Data were presented as means ± SEM (n = 9). (**b**) Quantification of cellular ROS levels via the fluorescent DCFH-DA assay in the *S*. *cerevisiae* WT and yeast mutants ∆*sir2* or ∆*tor1*. Cells were treated with antioxidants 0.008 mg/mL of quercetin (Q), 0.002 mg/mL of protocatechuic acid (PCA) or 0.1 mg/mL Hom Dang rice bran extract (HD). After, cells were exposed to 5 mM of hydrogen peroxide (H_2_O_2_) for 1 hr. to induce generation of ROS. Endogenous ROS levels of cells were measured. Error bars represented standard error of the mean (SEM) (*p, ^#^*p* < 0.01, two-tailed Student’s t test compared to untreated condition and wild-type, respectively). (**c**) The oxidative stress resistance of the WT, the ∆*sir2* and the ∆*tor1* strains were examined via spot assays. Cells were first treated or untreated with antioxidants quercetin (Q), protocatechuic acid (PCA) or Hom Dang rice bran extract (HD), and then exposed to 5 mM hydrogen peroxide (H_2_O_2_) for 1 hr. prior to be spotted on YPD plates. Growth was examined after incubation in a dark room at 30 °C for 48 hr. Three independent experiments were done. (**d**) Quantification of intracellular ROS levels in the chronological ageing *S*. *cerevisiae* WT, the ∆*sir2* and the ∆*tor1* strains treated with antioxidants 0.008 mg/mL of quercetin (Q), 0.002 mg/mL of protocatechuic acid (PCA), or 0.1 mg/mL Hom Dang rice bran extract (HD). The ROS levels were analyzed by the fluorescent DCFH-DA assay. Error bars represented standard error of the mean (SEM) (*p, ^#^*p* <0.01, two-tailed Student’s t test compared to untreated condition and wild-type, respectively).

Sir2 regulates NAD-dependent deacetylase, which participates in a wide range of cellular events, including chromosome silencing, chromosome segregation, DNA recombination and the determination of life-span^32^. It is also involved in the transcriptional repression of the silent mating-type loci, HML and HMR, and telomeric silencing via the association with Sir3 and Sir4^33^. First, the ROS detection experiments and spot assays were performed to identify the oxidation states of cells and to test whether the long-lived ∆*sir2* strain is oxidative stress resistant. The wild-type and the ∆*sir2* strains were either pre-treated or untreated with antioxidants Q and PCA or Hom Dang rice bran extract (HD) and then exposed to 5 mM hydrogen peroxide (H_2_O_2_) for 1 hr. The untreated ∆*sir2* strain showed higher ROS accumulation when compared to the untreated wild-type strain by approximately 45% (Fig. 2a). Pre-treatment with antioxidants Q or PCA or the extract (HD) by approximately 22%, 0%, or 56%, respectively, decreased the ROS accumulation in the ∆*sir2* strain and optimized growth (Fig. 2a,b). Following the exposure to H_2_O_2_, the highest ROS level and increased sensitivity was found for the ∆*sir2* strain by approximately 21% (Fig. 2a,b). The antioxidants Q, PCA and especially the Hom Dang rice bran extract (HD), could lower the ROS levels of the ∆*sir2* strain by approximately 34%, 4%, or 54%, respectively, and showed improved growth on the spot tests (Fig. 2a,b). Thus, the ∆*sir2* strain had high intracellular ROS accumulation and showed increased sensitivity to H_2_O_2_ while the antioxidants pre-treatment reduced ROS accumulation and enhanced the growth of the ∆*sir2* strain during the oxidative stress (Fig. 2a,b).

Next, the oxidation states of ∆*sir2* mutant pre-treated or untreated with the antioxidants Q or PCA or the Hom Dang rice bran extract (HD) were investigated to examine the possible correlation between antioxidants and longevity. Deletion of *SIR2* resulted in increased percent cell survival and extension of life-span by 2 days at 50% viability when compared to the untreated wild-type strain (Fig. 2c; dash black line versus solid black line) or approximately 65% at day 17. In contrast to the wild-type *S*. *cerevisiae* strain with extended life-span of approximately 179–205%; no life-span extension was found in the antioxidant pre-treated ∆*sir2* strain (Fig. 2a; dash red lines versus dash black line at day 17). To examine the oxidation state of the aging ∆*sir2* strain in the pre-treatment/un -treatment with antioxidants or the extract, the ROS levels were again measured as cells chronologically aged. As shown, the ROS levels were increased as cells aged (measured from day 0 to day 17) for both the wild-type and ∆*sir2* strains by approximately 71% and 68%, respectively (Fig. 2d). Between days 3–11, in untreated condition, the ROS levels of the ∆*sir2* strain were higher than those of the wild-type strain, suggesting elevated intracellular oxidative stress in cells lacking Sir2 (Fig. 2d). Absence of Sir2 function has been reported to result in a complete loss of transcriptional silencing, an increase in the rate of rDNA repeat and recombination, and a decrease in chromosome stability, leading to defects in the meiotic pachytene checkpoint, which may explain the observed increasing^1,4,34,35^.

Quercetin (Q), protocatechuic acid (PCA), and the extract of Hom Dang rice bran (HD) greatly reduced ROS levels in the wild-type and ∆*sir2* strains, especially during day 3–7 to approximately 45–96%, respectively, in WT (Fig. 2d and Table 4). However, ROS levels remained quite high between days 11 and 17 as cells aged, despite their antioxidant property (Fig. 2d). Despite elevated ROS levels in the ∆*sir2* strain, its longevity was extended (Fig. 2c,d). Pre-treatment with the antioxidants Q or PCA reduced ROS levels in both the wild-type and ∆*sir2* strains, but only enhanced the longevity of the wild-type strain but not ∆*sir2* strain (Fig. 2c,d). No clear correlation between the ROS levels, antioxidants and longevity could be found for the ∆*sir2* strain as no life-span extension was observed upon antioxidant pre-treatment despite a reduction in the ROS level (Fig. 2c,d). Thus, results suggested that the antioxidants could partly reduce ROS levels of the ∆*sir2* strain.

**Table 4:** Normalized ROS levels and difference in ROS levels normalized to day 0, the untreated condition or WT of CLS assay at day 0, 7, 13, and 17 for the ∆*sir2* and ∆*tor1* strains. Data used in comparison were significant at *p* < 0.001. Q, PCA and HD referred to quercetin, protocathechuic acid and Hom Dang, respectively. Negative values indicated the decreased values.

Strain	Day	% Normalized ROS levels compared to day 0				% Difference in ROS levels normalized to day 0				% Difference in ROS levels normalized to untreated condition				% Difference in ROS levels normalized to WT			
		untreated	Quercetin (Q)	PCA	HD extract	untreated	Quercetin (Q)	PCA	HD extract	untreated	Quercetin (Q)	PCA	HD extract	untreated	Quercetin (Q)	PCA	HD extract
WT	0	100	100	100	100	0	0	0	0	0	−91	−84	−89	0	0	0	0
	3	202	279	319	487	50	64	69	79	0	−88	−75	−73	0	0	0	0
	5	261	356	383	587	62	72	74	83	0	−88	−77	−75	0	0	0	0
	7	294	536	489	705	66	81	80	86	0	−84	−74	−73	0	0	0	0
	11	304	1537	1023	1545	67	93	90	94	0	−56	−48	−43	0	0	0	0
	13	324	2044	1429	1998	69	95	93	95	0	−45	−32	−31	0	0	0	0
	17	340	2653	1685	2293	71	96	94	96	0	−32	−23	−24	0	0	0	0
∆*sir2*	0	100	100	100	100	0	0	0	0	0	−87	−84	−81	10	63	13	85
	3	219	68	237	256	54	−47	58	61	0	−96	−83	−78	19	−60	−16	−3
	5	277	635	441	407	64	84	77	75	0	−70	−75	−72	17	192	30	28
	7	290	1097	995	802	65	91	90	88	0	−51	−45	−48	8	234	130	110
	11	300	1418	1143	996	67	93	91	90	0	−39	−39	−37	9	51	26	19
	13	301	1609	1435	1216	67	94	93	92	0	−31	−24	−24	2	29	13	13
	17	313	1837	1529	1312	68	95	93	92	0	−24	−22	−21	1	13	3	6
∆*tor1*	0	100	100	100	100	0	0	0	0	0	−90	−83	−81	12	22	23	94
	3	181	228	260	252	45	56	61	60	0	−88	−75	−73	0	0	0	0
	5	277	1141	554	629	64	91	82	84	0	−61	−66	−56	19	292	78	108
	7	282	1403	883	790	65	93	89	87	0	−53	−47	−45	7	220	122	117
	11	296	1974	1040	951	66	95	90	89	0	−37	−40	−37	9	57	25	19
	13	299	2239	1350	1201	67	96	93	92	0	−29	−23	−22	3	34	16	16
	17	311	2551	1489	1325	68	96	93	92	0	−22	−18	−17	2	18	9	12

We reported the identification of a Sir2-independent pathway responsible for the longevity extension associated with antioxidants. Besides, Sir2 and additional mechanisms such as antioxidative defense, which remains intact in the ∆*sir2* strain may operate to reduce the cellular ROS accumulation, may act in parallel pathways to promote longevity in yeast and, perhaps, higher eukaryotes. The higher ROS level observed in the ∆*sir2* strain, as a resulting of cellular activity, may not be harmful to cells but instead protective. Previous work has shown that severe calorie restriction or additional mutations are required to extend the life span of the ∆*sir2* mutant^34^. Increasing ROS level may be responsible for enhanced longevity in the *SIR2* deletion strain through activation of the antioxidative system although additional of excessive oxidative stress from (H_2_O_2_) exposure may be dangerous. Nevertheless, the Hom Dang rice bran extract showed promising anti-aging property and could replace chemically synthesized pure antioxidants used for ROS reduction and longevity enhancement at a lower cost.

### Anti-aging effect and involvement of Tor1 pathway

The Tor1 kinase controls several cellular processes to regulate cell cycle progression from G1 to S phase according to environmental signals^36^. Again, the oxidative stress resistance and spot assays were repeated by exposing the antioxidant-treated and untreated ∆*tor1* strain to 5 mM hydrogen peroxide for 1 hr. The untreated ∆*tor1* strain showed the highest ROS level as observed for the ∆*sir2* strain (Fig. 2a). This increased ROS level correlated to the observed increased sensitivity (Fig. 2b). The antioxidants and Hom Dang rice bran extract (HD) could reduce the ROS levels of the ∆*tor1* strain by approximately 37%, resulting in better growth compared to the untreated ∆*tor1* strain (Fig. 2a,b). Similarly, these antioxidants could only partly reduce ROS levels as observed. The examination of cell survival, using a spot test, revealed that regardless of increasing ROS level, deletion of *TOR1* could overcome the requirement for antioxidant pre-treatment to extend longevity (data not shown). It appeared that increasing ROS level may activate other pathways important for the oxidative defense system. In fact, the cellular function regulated by Tor1 has been shown to contain a general mechanism involving the sequestration of the oxidative stress response transcription factors Msn2/Msn4^37^. Examination of these factors may provide better understanding of the connection between Tor1-signalling pathways, aging, and oxidative stress response.

Similar treatments were performed using *S*. *cerevisiae* strain with a deletion of *TOR1* gene. The ∆*tor1* strain exhibited increased percent survival and could extend the life-span by 2 days at 50% viability when compared to the wild-type cells under untreated condition (Fig. 2c; dash black versus solid black line). While the antioxidants protocatechuic acid (PCA) and quercetin (Q), or the Hom Dang rice bran extract (HD) further extended the longevity of the wild-type strain, they could not show the same effect in the ∆*tor1* strain (Fig. 2c; dash red versus solid red line and solid blue and green line versus solid black line). The ∆*tor1* strain pre-treated with the Hom Dang rice bran extract (HD) did not show the life-span extension when compared with the untreated strain (Fig. 2c; dash red versus solid red line). In addition, the ∆*tor1* strain pre-treated with PCA, Q or the HD extract could no longer extend its longevity further (Fig. 2c; dash blue and green line versus dash black line). To examine the oxidation state of the aging ∆*tor1* strain, the ROS levels of cells were again measured for a period of 17 days. Under untreated condition, the ∆*tor1* strain showed similar ROS level as that of the wild-type strain except for a slightly higher ROS level at day 5 (Fig. 2d). Quercetin (Q), protocatechuic acid (PCA), and Hom Dang rice bran extract (HD) could reduce the ROS levels of ∆*tor1* strain by approximately 17–22% at day 17, although the ROS level remained higher than that of the wild-type strain under this condition (Fig. 2d). This indicated that the longevity effects of the Hom Dang rice bran extract and antioxidants resembles to Tor1 deletion in life-span extension.

### Roles of longevity factors in oxidative stress resistance

**Figure 3:**
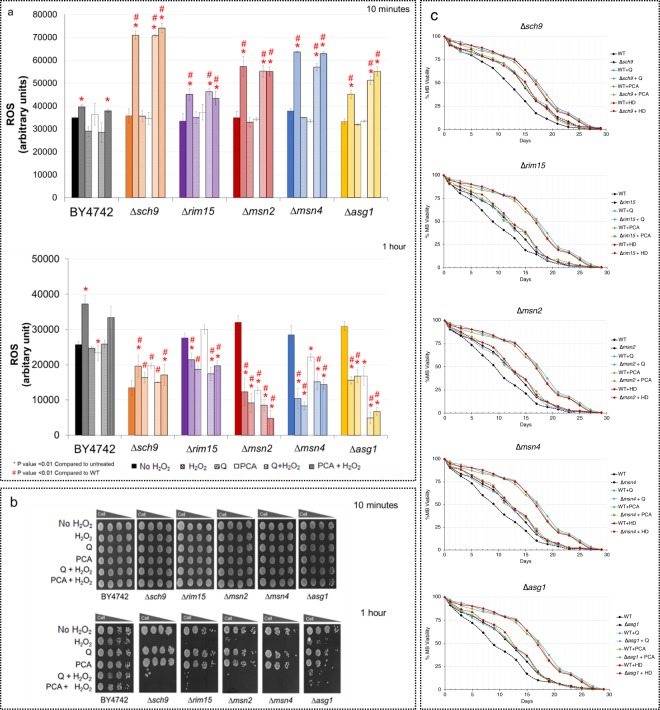
(**a**) Quantification of cellular ROS levels via the fluorescent DCFH-DA assay in the *S*. *cerevisiae* WT and yeast mutants ∆*sch9*, the ∆*rim15*, the ∆*msn2*, the ∆*msn4* and the ∆*asg1* strains. Strains were treated with antioxidants 0.008 mg/mL of quercetin (Q), 0.002 mg/mL of protocatechuic acid (PCA) or 0.1 mg/mL Hom Dang rice bran extract (HD). After, cells were exposed to 5 mM of hydrogen peroxide (H_2_O_2_) for 10 mins (upper panel) or 1 hr (lower panel) to induce generation of ROS. Endogenous ROS levels of cells were measured. Error bars represent standard error of the mean (SEM) (*p, ^#^*p* < 0.01, two-tailed Student’s t test compared to untreated condition and wild-type, respectively). (**b**) The oxidative stress resistance of the WT, the ∆*sir2* and the ∆*tor1* strains were examined via spot assays. Cells were first treated or untreated with antioxidants quercetin (Q), protocatechuic acid (PCA) or Hom Dang rice bran extract (HD), and then exposed to 5 mM hydrogen peroxide (H_2_O_2_) for 10 mins (upper panel) or 1 hr (lower panel) prior to be spotted on YPD plates. Growth was examined after incubation in a dark room at 30 °C for 48 hr. Three independent experiments were done. (**c**) The life-spans of yeast deletion mutants lacking longevity factors Sch9, Rim15, Msn2, Msn4 or Asg1 in the presence and absence of antioxidants quercetin (Q), protocatechuic acid (PCA) or Hom Dang rice bran extract (HD). Survival curves of chronologically aging yeast cells from day 0 to day 30 were shown. Lack of Sch9 significantly increased the longevity while, in contrast, lack of Rim15, Msn2, Msn4 or Asg1 significantly decreased the longevity of *S*. *cerevisiae*. Pretreatment with antioxidants quercetin (Q) or protocatechuic acid (PCA) or Hom Dang rice bran extract (HD) showed increasing life-span extension in the WT, the ∆*rim15*, the ∆*msn2*, the ∆*msn4* and the ∆*asg1* strains. (*p, ^#^*p* < 0.01, two-tailed Student’s t test compared to untreated condition and wild-type, respectively). Data were presented as means ± SEM (n = 9).

**Figure 4:**
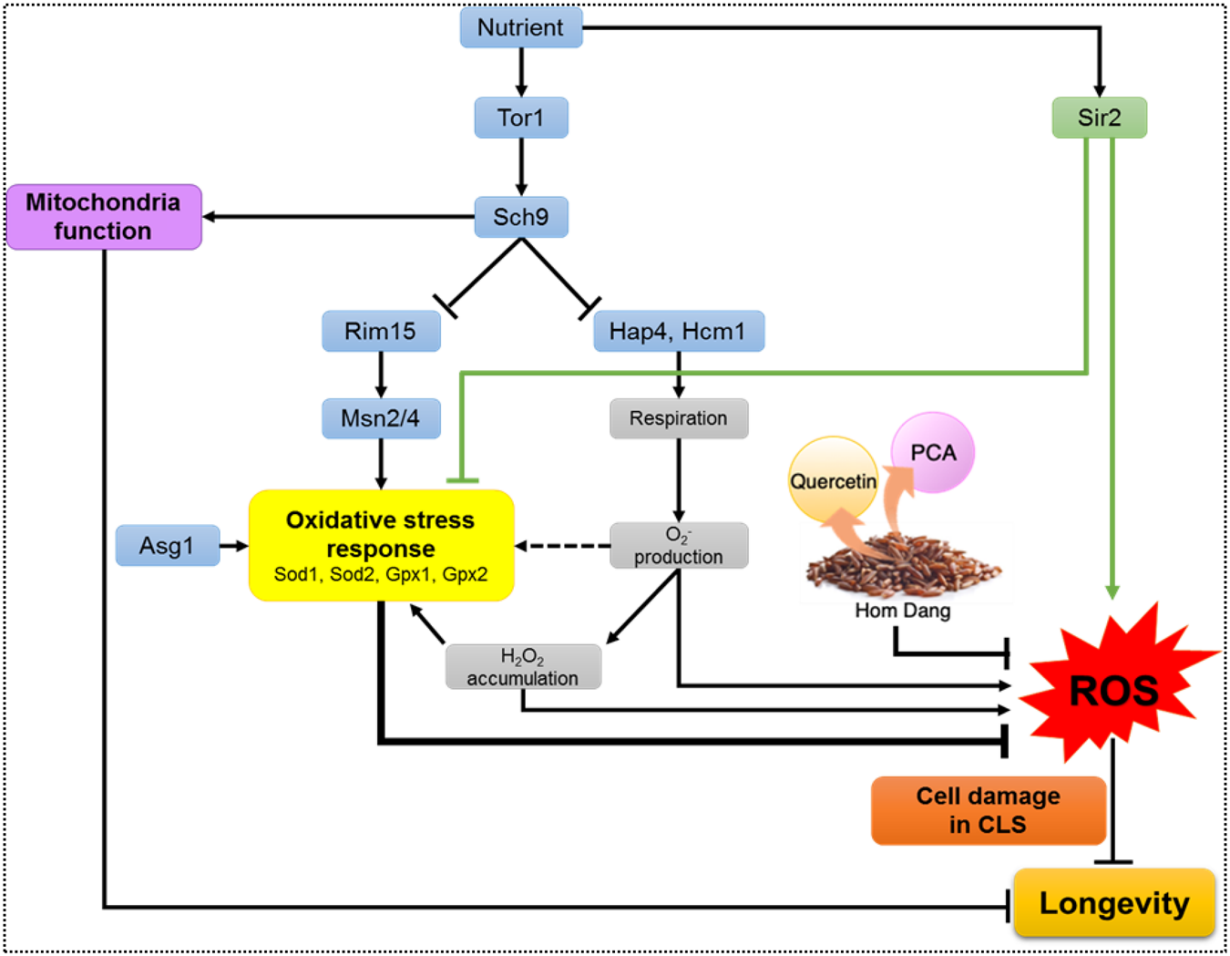
Proposed model to describe modes of actions of antioxidants quercetin (Q), protocatechuic acid (PCA) or Hom Dang rice bran extract (HD) to reduce intracellular ROS accumulation and roles of longevity factors of the Tor1- and Sir2-signaling pathways and transcription factors of the oxidative stress response system to reduce or promote longevity in the model yeast *S*.*cerevisiae*.

Again, the ROS levels of strains lacking longevity kinases Sch9 or Rim15, and transcription factors Msn2, Msn4 or Asg1 were examined following treatment with antioxidants and then with hydrogen peroxide (H_2_O_2_) for a duration of 10 min or 1 hr. As shown, these mutants showed rapid elevated ROS levels after a 10-min exposure to H_2_O_2_ (Fig. 3a; upper panel). Treatments with quercetin (Q) or protocatechuic acid (PCA) did not reduce the ROS levels (Fig. 3a; upper panel). Examination of cell survival via spot assays showed that the short exposure to H_2_O_2_ (10 min) was not able to inhibit the growth of these deletion mutants of oxidative stress response and in turn induced ROS levels which may immune the cells for better tolerance to oxidative stress (Fig. 3b; upper panel). Also, cells remained viable as there could be a functional redundancy in activating the stress response due to a single gene deletion. Surprisingly, for the longer H_2_O_2_ exposure of 1 hr., the ROS levels were reduced greatly, and treatments with antioxidants further decreased the ROS levels (Fig. 3a; lower panel). The observed lower ROS levels were likely due to the inactivation of enzymes for the conversion of the ROS signal to a fluorescence signal as a result of cell death. Since, inactivation of Sch9 may cause up-regulation of many stress-resistance and DNA-repairing genes as well as genes required to extend the chronological lifespan. In fact, the ROS level should be very high under this condition. The long H_2_O_2_ exposure was found to be detrimental for all tested strains, and addition of antioxidants quercetin (Q) or protocatechuic acid (PCA) or the Hom Dang rice bran extract (HD) slightly promoted growth for some strains, such as the ∆*asg1*, ∆*rim15*, and ∆*sch9* strains (Fig. 3b; lower panel). The correlation between the oxidation state of these mutants and their roles in mediating oxidative stress response was clear. Antioxidant pre-treatment resulted in the lowering ROS levels in these deletion mutants the ∆*rim15*, the ∆*msn2*, the ∆*msn4* and the ∆*asg1*, suggesting that the oxidative stress defense system is a major route for maintaining the cellular ROS level (Fig. 4). The correlation between the oxidation states and longevity of these mutants remains to be further investigated. Likely, their ROS levels would be high due to their direct roles in controlling the oxidative stress response (Fig. 3a) and cellular homeostasis, as observed for shortened longevity of these mutants (Fig. 3c).

### Downstream kinases and transcription factors of longevity in *S*. *cerevisiae*

Notably, similar effect was observed for the downstream kinases of Tor1 such as Sch9 but not Rim15. Deletion of *SCH9* resulted in increased percent cell survival; however, deletion of *RIM15* decreased percent cell survival by 2 and 3 days at 50% viability, respectively when compared to the untreated wild-type strain (Fig. 3c). Unlike the wild-type strain (Fig. 3c), antioxidants quercetin (Q) and protocatechuic acid (PCA) or Hom Dang rice bran extract (HD) could not extend the longevity of the ∆*sch9* strain, but could extend that of the ∆*rim15* strain (Fig. 3c). Msn2 and Msn4 are positive transcriptional factors that act as a component of the stress responsive system. They recognize and bind to the stress response element (STRE) in response to various forms of stress (heat, oxidative, osmotic shock and others)^35,38,39^. Deletion of *MSN2* or *MSN4* genes exhibited decreased percent cell survival by 3 days at 50% viability when compared to the untreated wild-type strain (Fig. 3a; dash black versus solid black line). The ∆*msn2* or ∆*msn4* strain pre-treatment with quercetin (Q), protocatechuic acid (PCA), or the Hom Dang rice bran extract (HD) showed an increase in life-span extension by approximately 78–121%, 78–127%, and 179–205%, respectively, as compared to the wild-type treated strain at day 17 (Fig. 3c; dash blue, green and red lines versus solid black line). Lastly, Asg1 is a recently characterized zinc cluster transcription factor of stress response^40^. Null mutant *asg1* has a respiratory deficiency, calcofluor white sensitivity and slightly increased cycloheximide resistance^41^. Deletion of *ASG1* also exhibited reduced percentage cell survival by 3 days at 50% viability when compared to the untreated wild-type strain (Fig. 3a; dash black versus solid black line). Likewise, the ∆*asg1* strain supplemented with tested antioxidants or Hom Dang rice bran extract (HD) could increase the life-span by approximately 78–121% at day 17 (Fig. 3a; dash blue, green and red lines versus solid black line).

## Discussion

Rice bran is produced as an agricultural byproduct of white rice processing. This nutrient-rich outer hull of rice kernel offers a number of health benefits as an excellent source of essential nutrients including antioxidants, vitamins, minerals, and fiber. The concentration of bioactive compounds and antioxidants present in rice bran or other cereal grains is significantly dependent on the grain color^22^. Among cereal grains, rice bran has more phenolic content and antioxidant activity than wheat, sorghum, corn and barley that could be applied in food and health industries^42^. The TPC of red and black rice bran extracts correlated well with the antioxidant activities obtained via the DPPH, ABTS radical scavenging ability, and Ferric ion reducing power (Table 1). The reported antioxidant activity could be attributed to the different kinds of antioxidants in the pigmented rice bran, as has been reported by other studies^35,38^, and could promote human health by reducing the concentrations of reactive cell-damaging free radicals. They act as antioxidants as singlet oxygen quencher and radical scavenger^39^.

The protective effects of protocatechuic acid and quercetin against oxidative stress via a direct antioxidant mechanism through free radical-scavenging activity and their anti-inflammatory activity have been documented^43^. For example, phenolic acids including protocatechuic acid, found only in red and black rice bran, and *p*-coumaric acid, found only in red rice bran, function as antioxidants and convert the superoxide anion to H_2_O_2_ by hydrogen donation and oxidization of hydroxyl radical to water^14^. This study shows that extracts of red and black rice bran restore the viability of yeast cells lacking the antioxidant enzyme, superoxide dismutases and glutathione peroxidases (Fig. 1a). The Hom Dang rice bran extract, quercetin and protocatechuic acid, also decrease ROS levels in the yeast cells up to day 21 after pre-treatment, implicating the anti-aging intervention (Fig. 1b). These results are consistent with previous studies, which confirm that proanthocyanidin found in Hom Dang rice bran (Table 2) increases the antioxidant capacity by stimulating the enzymatic activities of superoxide dismutase and catalase^44^.

Also, Hom Dang rice bran contains compounds with antioxidant and anti-inflammatory activities in accordance with the inhibition of protonation of the superoxide anion and the hydroxyl radical initiation of lipid peroxidation, which support the finding that these antioxidants could help to restore damaged cell membranes (Fig. 1d). The previous report also showed that Hom Dang rice bran extract can scavenge the superoxide anion and hydroxyl radicals due to its bioactive compounds contents^45^. These results well corroborate a previous finding that quercetin significantly suppresses the level of malondialdehyde, a naturally end product of lipid peroxidation and an oxidative stress biomarker, which could contribute to the protective effect on cell membranes^42^. In addition, quercetin found in the Hom Dang rice bran (Table 2) has been shown to act as estrogenic agonist which activates estrogen activity to inhibit cell proliferation in human breast cancer^46^. Therefore, rice bran extract has a wide spectrum of health benefits and pharmacological functions^27,47,48^. Some medical studies have also revealed that high consumption of whole grain could reduce the risk of coronary disease^35^. Protocatechuic acid could be spontaneously degraded by microbial colon and function as anti-colorectal cancer^49^. Some studies have claimed that phenolic compounds from rice bran found in the intestine act to promote enzymatic digestion and absorption of nutrients, thereby preventing colorectal cancer and chronic diseases^50^.

Due to mammalian analogy, *S*. *cerevisiae* is widely used as a model for aging studies to elucidate new targets and cellular mechanisms^51^. A number of antioxidants and natural compounds, including resveratrol, quercetin, and tanshinone, has been shown to extend the life-span of *S*. *cerevisiae*^52–54^. The increase in cell viability under stress conditions has been linked to longevity^14^. Here, antioxidants found in pigmented rice extracts (Table 2) and some signaling and stress responsive factors are shown to display longevity effect in the yeast model of ageing (Fig. 3). These important factors determine life-span extension in a very complex fashion. The extract of Hom Dang rice bran or antioxidants quercetin or protocatechuic acid delays chronological aging and extends the life-span significantly via a reduction of the ROS accumulation (Figs. 2 and 3). The stress responsive kinases and transcription factors play an important role in altering ROS accumulation (Figs. 2 and 3). With respect to longevity promotion, in addition to its anti-oxidative property, antioxidants may influence longevity through modulation of the expression of target genes related to *SIR2* of the sirtuin family or the TOR pathway, as is the case in mammals^55,56^. They may directly or indirectly function by activating the Rim15-mediated stress response system to enhance the production of antioxidant enzymes for reduction of ROS level (Fig. 4). Further investigation will be required. Nevertheless, the results suggest that Hom Dang rice bran extract and the antioxidants mimic the inactivation effect of Sir2, Tor1 or Sch9 for reduction of ROS level and compensate for the activation of stress response system as shown for Rim15, Msn2, Msn4 or Asg1 deletion (Fig. 4).

The antioxidant pre-treatment promotes longevity in the absence of Rim15 kinase. Tor1 kinase activates Sch9 and functions to inhibit Rim15. In addition, inactivation of Sch9, a homolog of the mammalian threonine protien-kinase Akt, increases stress resistance and extends the chronological life span of non-diving cells through activation of respiration via Hap4 and Hcm1^37,57^.

This leads to the production of superoxide radicals that are further converted to hydrogen peroxide whose accumulation then signals to activate the stress response system for the reduction of ROS and cell damages thereby increasing life-span extension (Fig. 4). Sch9 also plays another important role in mediating mitochondrial functions which affects oxidative stress and is related to the chronological life-span (Fig. 4). The accumulated ROS may activate downstream components of Rim15, including transcription Msn2/4 and Asg1, for activation of antioxidant enzymes associated with longevity^19^ (Fig. 4). Importantly, this study identifies a new role of the transcription factor Asg1. It not only functions in stress response but also acts as a longevity factor as observed for the Msn2/4 transcription factors of the TOR signaling pathway (Fig. 4). Together, they activate the expression of antioxidant enzymes to decrease ROS accumulation and restore cellular damages during exposure to oxidative stress (Fig. 4). In addition, Asg1 is required for full activation of genes in several pathways of fatty acid synthesis, including β-oxidation (*POX1*, *FOX2*, and *POT1*) and triacylglycerol breakdown (*TGL3*), which affect cellular lipid contents and plasma membrane permeability^44^. As shown, plasma membrane permeability could be preserved in strains pre-treated with the extract or antioxidants (Fig. 1d). Further the roles of these new longevity factors in life-span extension, particularly in relation to lipid metabolism, need to be explored.

In addition to tested compounds, it has been reported that protocatechuic acid and proanthocyanidin are able to elevate catalase and superoxide dismutase activities, and treatment with rutin present in red rice bran (Table 2) could elevate the transcription levels of genes encoding MnSod and catalase, while reducing the transcription level of Tor^22^. Furthermore, catechin present in the red rice bran (Table 2) also acts as a powerful hydrogen-donating radical scavenger of ROS and RNS and chelates divalent transition metal ions (Cu^2+^, Zn^2+^ and Fe^2+^), thereby preventing the Fe^2+^-induced formation of free radicals^58^. It also increases the expression of manganese superoxide dismutase *SOD2* gene in *Drosophila melanogaster* and inhibits the signaling of transcription factor NF-*κ*B, leading to reductions in liver and kidney damage, and improvement of age-associated inflammation and oxidative stress in mice^45^. Thus, the evidence provided here and in other studies clearly support the antioxidative-assisted anti-aging effects of the pigmented rice extract and antioxidant compounds.

The discovery of new natural antioxidants with longevity effect could offer advantageous strategies for the protection against harmful radicals, chronic inflammation, and age-related diseases^59,60^. It is, therefore, necessary to explore ways to utilize agricultural by-products, such as pigmented rice bran, as food, dietary supplements, or healthcare products to promote our health and overall well-being^27,47,48^. Further investigation in yeast may serve as a useful model to elucidate conserved mechanisms of aging and age-related diseases in humans.

## Materials and Methods

### Materials

The two Thai pigmented rice cultivars, namely Hom Dang (KMDL105R-PSL-E-14) and Kum Doi Saket (KKU2012-PS-PANPB-OS-001), were kindly supplied by S&J International Enterprises Public Co., Ltd., Bangkok, Thailand. The following chemicals were used for the preparation of yeast growth medium (YNB) 1% yeast extract (Aldrich-Sigma, USA), 2% peptone (Aldrich-Sigma, USA), 2% glucose (Aldrich-Sigma, USA), and 2%agar (Aldrich-Sigma, USA). *S*. *cerevisiae* mutant strains: BY4742 (WT) and single deletion strains obtained from Open Biosystem (Dharmacon, Inc., Lafayette, CO, USA).

### Microbial and treatments

The yeast cells were grown in the synthetic minimal YNB medium initially containing 2% glucose, in the presence of quercetin 0.008 mg/mL (Aldrich-Sigma, USA), protocatechuic acid 0.002 mg/mL (Aldrich-Sigma, USA), and Hom Dang rice bran extracts concentration at (1, 0.1, 0.01 and 0.001 mg/mL) (S&J International Enterprises Public Company Limited).

### Determination bioactive compounds by UPLC MS/MS

An ultraperformance liquid chromatography (UPLC), Waters Acquity (Milford, MA, USA), system was coupled with a triplequadrupole-linear ion trap tandem mass spectrometer (Applied Biosystems 4000 Q TRAP; Life Technologies Corporation, Carlsbad, CA, USA) with an electrospray ionisation (ESI) source. A C_18_ reversed phase Acquity column (1.7 *μ*m, 150 mm × 4.6 mm). The mobile phase was a binary solvent system consisting of solvent A (water with 0.1% formic acid) and solvent B (CH_3_CN). The UPLC gradient for mass screening was 0–5 min, 90% A; 5–15 min, 90–10% A; 15–20 min, 10% A; 20–25 min, 10–90% A; 25–30 min, 90% A. The flow rate was 0.25 mL/min, and the injection volume was 10 *μ*L. All samples were filtered with 0.2 *μ*m nylon membrane filter prior to injection. The mass spectra were acquired from *m*/*z* 100–1000 with a 20 ms ion accumulation time. All mass spectrometric data were acquired in positive ionisation mode. The capillary and voltage of the ESI source were maintained at 400 °C and −4.5 kV, respectively. The scan rate was 1000 amu/s. Data acquisition and data processing was performed using Analyst 1.4.2. MS Fragmenter 12.0 (Advanced Chemistry Development, Toronto, Canada) was used to predict compound fragmentation^61^.

### Determination of total phenolic content (TPC)

The total phenolic content of each fraction was determined using the Folin-Ciocalteu method with some modifications. Briefly, 50 µl of extract solution was shaken for 1 min with 1 mL of diluted (1:10 with water) Folin-Ciocalteu reagent. After the mixture was shaken, 50 µL of 10% Sodium carbonate (Na_2_CO_3_) was added, and the final volume was made up to 5 mL with distilled water. After 2 hour of reaction, the absorbance at 760 nm was determined and used to estimate the phenolic acid content using a standard curve prepared using gallic acid^61^.

### Free-radical scavenging activity on DPPH

Total free radical scavenging capacity of the extracts from two pigmented rice bran extracts: red and black was estimated according to the previously reported method with slight modification using the stable DPPH radical, which has an absorption maximum at wavelength of 517 nm. All the determinations were performed in triplicate. The capability to scavenge the DPPH radical was calculated using the following equation. DPPH Scavenged (%) = (A_517_ of sample- A_517_ of control/A_517_ of control) × 100 (1), where, A_517_ of control is absorbance at t = 0 min; A_517_ of sample is absorbance at t = 30 min.^61^.

### 2,2′-azino-bis-3-ethylbenzthiazoline-6-sulphonic acid cation decoloration (ABTS) assay

2,2′-azino-bis-3-ethylbenzthiazoline-6-sulphonic acid (ABTS) assays was prepared by mixing ANTS (7 mM) with potassium persulfate (2.45 mM) in volume ratio of 1:1. The solution was stored in dark condition 6 hours, and dilute with ethanol to the absorbance of 0.7 ± 0.02 at 734 nm. The experiment was carried out by 0.2 mL extract to 1.8 mL of diluted ABTS• + solution. The absorbance was recorded after 20 minutes incubation. The affinity of sample to quench free radical was evaluated. The results were expressed as value of IC_50_. All samples were tested in triplicate. The calculation is stated as scavenging % = [(A of sample-A of control)/A of control] × 100%^61^.

### Ferric reducing antioxidant power activity (FRAP)

The chelating capacity of extracts measured using ferrous ion (Fe^3+^) reduction assay. Sample (0.1 mL) with the concentration range from 20–100 µg/mL was dissolved in 2.5 mL of sodium buffer (0.2 M, pH 6.6) before adding 1% w/v potassium ferricyanide III (2.5 mL). After the mixture was incubated at 50 °C for 20 minutes, 2.5 mL trichloroacetic (10% w/v) was added into the mixture. The upper layer of solution (2.5 mL) was diluted with distilled water (2.5 mL) and then added with iron(III)chloride (0.1% w/v) 500 µL then measure at 700 nm^31,61^.

### Spot assay

WT and mutant strains (the Δ*sod1*, Δ*sod2*, Δ*gpx1*, Δ*gpx2*, Δ*ctt1*, Δ*cta1*, Δ*tor1*, Δ*sir2*, Δ*sch9*, Δ*rim15*, Δ*msn2*, *Δmsn4* or Δ*asg1* strains) were first grown overnight. After, cells were regrown to OD_600_ of 0.6., 0.001, 0.01, or 0.1 mg/mL pigmented rice bran extract or (0.008 mg/mL) quercetin (Q), (0.002 mg/mL) protocatechuic acid (PCA) were then added to the mixture of 100 ul of cells, followed by the exposure to 5 mM H_2_O_2_ for 10 mins. or 1 hr. at 30 °C before being spotted on YPD agar medium and incubated in a dark room at 30 °C for 48 hr.

### Dichloro-dihydro-fluorescein diacetate (DCFH-DA) assay for ROS detection

Endogenous ROS levels of *S*. *cerevisiae* cells were measured by a fluorometric assay using 2′,7′-dichlorofluorescein diacetate (DCFH-DA) (Sigma-Aldrich) as a ROS indicator, as previously described ^62^. For chronological study, WT, Δ*tor1* and Δ*sir2* strains were inoculated overnight then diluted to OD_600_ of 0.1. Antioxidants or the rice bran extract was added to the cell cultures at the final concentration of (0.008 mg/mL) quercetin (Q), (0.002 mg/mL) protocatechuic acid (PCA) or 0.1 mg/mL pigmented rice bran extract. Cells were incubated at 30 °C from day 0 at the OD_600_ of 0.6 to day 17. For the oxidative stress assay, WT, Δ*tor1*, Δ*sir2*, Δ*sch9*, Δ*rim15*, Δ*msn2*, Δ*msn4 or* Δ*asg1* strains were first pretreated with the antioxidants or the rice extract prior to be exposed to 10 mins. or 1 hr. of 5 mM H_2_O_2_ at 30 °C. Then, 5 mL of cell were treated with (30 µL of 10 mM) DCFH-DA and incubated at 37 °C for 30 minutes. The cells were harvested as described in^62^. ROS induction was measured using a fluorescent microplate reader (excitation 490 nm, emission 535 nm).The protein content of the samples was measured using Bradford assay, and the protein concentration used to normalize the ROS induction of each sample.

### Plasma Membrane Permeability assay

Propiodium Iodine (PI, Thermo Fisher Scientific, Taiwan) staining assay performed on yeast cells. Yeast strains were at to log phase in YPD. After cells were treated with (0.008 mg/mL) of quercetin, (0.002 mg/mL) of protocatechuic acid, (0.1 or 0.01 mg/mL) Hom Dang rice bran extract (HD) grown to day 1 or day 7. The supernatant was removed and, the cells were washed three times with PBS with 0.01% Tween 20 and then staining with PI solution (Sigma-Aldrich) at 5 µg/mL of final concentration and then incubated in the dark place at 37 °C for 30 minutes. The cells were washed 3 times and observed under the fluorescence microscope (IX83, Inverted Microscopes, Olympus, Japan)^62^.

### Chronological lifespan assay

Wild-type and mutants cells were grown in the synthetic minimal YNB medium initially containing 2% glucose in the presence or absence of extract or antioxidants quercetin or protocatechuic acid at the final concentrations of (0.1 mg/mL), (0.008 mg/mL) and (0.002 mg/mL), respectively. The cellular viability was determined by the methylene blue technique using Thomas’s counting chamber^51^. The number of stained (non-active cells) or un-stained (active cells) and the number of buds were counted in five different fields with a total of at least 200–300 cells. The percentage of ‘viable’ cells (%MB ‘viability’) was the number of un-stained cells (live cells) divided by the total number of cells (stained and unstained). Under the conditions used, mean viability was estimated with an accuracy of 10%^63^.

### Statistical analysis

Data are presented as means ± SEM (n = 9; *p < 0.01; the p values for comparing the means of two groups were calculated using the IBM SPSS statistics software using t-test). Also, data are presented as means ± SEM (n = 9; *p < 0.01; the *p* values for comparing)^63^.
